# “Everyone Should Be Able to Choose How They Get Around”: How Topeka, Kansas, Passed a Complete Streets Resolution

**DOI:** 10.5888/pcd11.130292

**Published:** 2014-02-20

**Authors:** Elizabeth A. Dodson, Marvin Langston, Lauren C. Cardick, Nancy Johnson, Paula Clayton, Ross C. Brownson

**Affiliations:** Author Affiliations: Marvin Langston, Saint Louis University School of Public Health, St. Louis, Missouri; Lauren C. Cardick, Ross C. Brownson, Washington University in St. Louis, St Louis, Missouri; Nancy Johnson, Heartland Healthy Neighborhood, Topeka, Kansas; Paula Clayton, Kansas Department of Health and Environment, Topeka, Kansas.

## Abstract

**Background:**

Regular physical activity can help prevent chronic diseases, yet only half of US adults meet national physical activity guidelines. One barrier to physical activity is a lack of safe places to be active, such as bike paths and sidewalks. Complete Streets, streets designed to enable safe access for all users, can help provide safe places for activity.

**Community Context:**

This community case study presents results from interviews with residents and policymakers of Topeka, Kansas, who played an integral role in the passage of a Complete Streets resolution in 2009. It describes community engagement processes used to include stakeholders, assess existing roads and sidewalks, and communicate with the public and decision-makers.

**Methods:**

Key informant interviews were conducted with city council members and members of Heartland Healthy Neighborhoods in Topeka to learn how they introduced a Complete Streets resolution and the steps they took to ensure its successful passage in the City Council. Interviews were recorded, transcribed, and analyzed by using focused-coding qualitative analysis.

**Outcome:**

Results included lessons learned from the process of passing the Complete Streets resolution and advice from participants for other communities interested in creating Complete Streets in their communities.

**Interpretation:**

Lessons learned can apply to other communities pursuing Complete Streets. Examples include clearly defining Complete Streets; educating the public, advocates, and decision-makers about Complete Streets and how this program enhances a community; building a strong and diverse network of supporters; and using stories and examples from other communities with Complete Streets to build a convincing case.

## Background

Leading an active lifestyle can be difficult given the infrastructure of many US cities. Residents in areas of distinct urban sprawl tend to walk less and weigh more than those living in more compact areas (1). Leisure walking is more prevalent in mixed-use neighborhoods than in purely residential neighborhoods (2). Increasing the number of sidewalks and biking paths in a community decreases pedestrian and bicycle injuries (3). Other environmental interventions and traffic-calming devices, such as pedestrian overpasses and refugee islands (ie, protected areas in the median on 2-way streets allowing pedestrians to cross in stages), can decrease pedestrian–vehicle crashes by 91% within 100 m of the overpass and reduce the risk of pedestrian–vehicle conflict by roughly two-thirds (3–5).

One policy strategy that can increase opportunities for walking and biking is the creation of Complete Streets, a program that advocates for streets designed to enable safe access for all users, including pedestrians, bicyclists, motorists, and transit riders of all ages and abilities as defined by the National Complete Streets Coalition (6). The Centers for Disease Control and Prevention (CDC) has cited the adoption of Complete Streets policies as an effective strategy for combating obesity, because people with access to safe walking spaces are more likely to meet national recommendations for physical activity (6,7).

## Community Context

### Topeka, Kansas

The Topeka metropolitan statistical area, with over 230,800 residents, is one of the largest in Kansas. This area comprises the city of Topeka (Shawnee County) and 5 surrounding counties. The median age of residents in Shawnee County is 38; surrounding counties have similar age distributions. Residents of Topeka aged 65 years or older account for only 13.8% of the total population (8).

The nationwide epidemic of obesity seems to be magnified in Topeka. According to CDC, over 32% of residents in this area were obese (body mass index [BMI] ≥30 kg/m^2^) in 2011 (9). This was higher than the prevalence of obesity in Kansas overall (29.6%) and nationwide (27.8%) and has steadily increased throughout Kansas over the past 14 years (9).

### Context of Complete Streets

The term “Complete Streets” was first used in December 2003 at a meeting of America Bikes and its partner organizations (10). The new term, born out of the concept of including bicyclists in transportation planning, was intended to be inclusive of pedestrians, public transit riders, and other road users (10). The Complete Streets Task Force (www.smartgrowthamerica.org/complete-streets/who-we-are) was formed in 2004 with the goal of including the first Complete Streets policy in a federal transportation bill; however, the task force soon refocused its efforts at state and local levels (10). Several pieces of legislation have been introduced at the federal level, most recently the Safe Streets Act of 2013 introduced in June 2013 by Representatives Doris Matsui (D-CA) and David Joyce (R-OH). If the legislation is enacted, this bipartisan effort would make Complete Streets a nationwide standard (11).

The objective of a Complete Streets program is to offer safe, accessible spaces for all road users, regardless of age or ability. In addition to improving safety, the adoption of Complete Streets policies has also been shown to improve health by encouraging walking and bicycling, to lower transportation costs for families, and to promote stronger communities through increased social engagement (6). Though no standardized format for Complete Streets exists, common elements include sidewalks, curb cuts, speed humps, and designated bike lanes (6).

In Topeka, a community-wide effort was undertaken to pass a local Complete Streets resolution. Our study focuses on the process by which the resolution was proposed and ultimately passed in Topeka, in November 2009. We interviewed key individuals to learn about the introduction of a local Complete Streets resolution and the steps taken to ensure its passage in the Topeka City Council. Our objective is to present these findings, share lessons learned, and make recommendations that may help other communities to enact a Complete Streets policy.

## Methods

This case study used a previously constructed questionnaire with revised sections to specifically address Complete Streets. Our research team pilot tested the questionnaire with collaborators from Topeka. We used 2 separate questionnaires to interview key informants, 1 designed for members of Heartland Healthy Neighborhoods (HHN), a local organization heavily involved in advocating for the passage of the Complete Streets resolution, and 1 for members of the Topeka City Council, which passed the resolution. Both questionnaires included 12 questions, 11 of which were open-ended.

### Data collection

Key informants included Topeka City Council members in office in November 2009 and HHN members identified by that organization’s leadership. Snowball sampling was used to identify additional people for interviews. Research staff made initial contacts with potential participants via telephone and conducted follow-up communication via e-mail. Up to 4 contact attempts were made per participant. The institutional review board of Washington University in St. Louis approved the study.

Trained staff administered the questionnaires by telephone interview in November 2010. Interviews were recorded and transcribed verbatim. Average interview length was 20 minutes, and participants were offered a $25 gift card for their time.

### Data analysis

Three independent coders used focused qualitative data analysis techniques to systematically analyze interview transcripts. The use of focused coding enabled coders to analyze transcripts by using the same set of thematic categories. Coders determined these categories jointly and in accordance with project aims. All transcripts were double coded to ensure accuracy. Only minimal discrepancies in coding were discovered; these were easily resolved, resulting in high interrater agreement.

## Outcome

A total of 18 interviews were completed, resulting in a 69% response rate. Findings from interviews are presented below and organized around key themes that emerged.

### Engaging the community to pass a Complete Streets resolution

Key informants interviewed reported that many events contributed to passage of the Complete Streets resolution in Topeka. The movement began in 2008, when the local Young Men’s Christian Association (YMCA) received a grant facilitating attendance at a training that included a Complete Streets presentation. A similar presentation had a powerful effect on the HHN leadership, which comprised diverse community leaders, who attended the training and gave them a strong desire to bring Complete Streets to Topeka. One participant noted,

It was at that meeting that I . . . had an almost revival experience in terms of the built environment and its impact on the health choices people make. . . . We became the core of advocating for Complete Streets . . . and it had a profound personal effect on me as well. I started walking and cycling . . . which really told me about the quality of our infrastructure.

Following the training, community members hired a consultant to evaluate the streets of Topeka for walkability and friendliness to all users. The evaluation was completed through an audit of existing streets and sidewalks to assess the presence or absence of features that facilitate safe transit for pedestrians and bikers (eg, sidewalks, curb cuts, crossing signals, bike lanes). The evaluation revealed that up to 90% of streets in Topeka were safe only for automobiles. Building on this information and the momentum from the training, members of HHN began in earnest to bring Complete Streets to Topeka. One critical action was HHN’s sponsorship of a 3-day workshop to introduce the idea of Complete Streets to the city and county. All potential stakeholders were invited to this workshop, including city engineers, city planners, elected officials, city council members, county commissioners, funders, and community members.

Several additional events helped build momentum for a Complete Streets movement. For example, physical activity advocates learned about Complete Streets at a conference. Cyclists formed a bicycle riding club. The death of a local cyclist drew attention to the issue. A clean indoor air campaign was undertaken in Topeka, and the city started planning for a downtown revitalization aimed at making Topeka an attractive destination for tourists and potential new residents.

One of the most important elements of success was the diverse HHN organization. The group included members from varied organizations, and thus, served as a central place of overlapping interests. As all of these events coalesced to facilitate passage of the Complete Streets resolution, 1 community member noted that “It was just like the perfect storm.”

### Opposition to Complete Streets

Key informants also described encountering opposition to their efforts to pass a Complete Streets resolution. Opposition came primarily from city traffic engineering staff, city streets and planning departments, and older taxpayers on a fixed income. As 1 participant noted,

At the beginning . . . the main resistance came from within the city streets and planning [departments] . . . and so it took a while for them to see a different way of doing business, a different way of looking at road design instead of just, “What’s the quickest way that we can move cars?” to more of a, “What’s the best way to move people?”

The primary reasons that the Complete Streets movement was opposed included community members’ struggles to understand what Complete Streets meant, fears about the cost of Complete Streets, a failure to see the preventive value of Complete Streets, and the struggling economy at the time. When describing opposition, 1 participant noted, “I just don’t think they saw the value . . . that Complete Streets has in terms of providing a safer environment and a more accessible environment for bicycling on the streets and walking and jogging in a safe environment. I just don’t think they caught the vision.”

### Support for Complete Streets

Supporters were far more numerous than those who opposed Complete Streets. Supporters came from various sectors including universities, the Metropolitan Planning Organization, the Topeka Chamber of Commerce, the county commissioner, community leaders, city council members, HHN members, the news media, the YMCA, the Kansas State Department of Health, and various exercise, bike, parks, and neighborhood coalitions. In fact, study participants noted that the diversity of support may have been one of the greatest strengths of this movement. Moreover, the leadership of the HHN was integral in moving the resolution along. As 1 participant noted,

[HHN is] probably my outstanding example of a highly functional community coalition that is just hitting on all cylinders, and they completely understand policy systems and environment work in terms of how that impacts the health of their residents. . . . They are inclusive. They . . . have a lot of insight, and not only that, but just flat out talent for bringing people together.

### Stories about Complete Streets

Storytelling was a particularly effective method that Topeka leaders used to share the message about Complete Streets with the community. Leaders used local, personal narratives to make their case. They used pictures of their community, roads, and sidewalks to illustrate the severity of conditions or to describe how local children were unable to cross streets to play because of the danger posed by unsafe roads and sidewalks.

Aside from telling stories about problems, leaders also enlisted people who had experienced the benefits of Complete Streets elsewhere and could testify to how well they worked and how much safer their communities had become. To further personalize their testimony, a group of HHN members began bike-commuting to fully experience biking in Topeka.

Some of the most powerful stories told involved the limitations faced by Topeka residents as they sought to be active in their communities:

A lot of times I’ll be riding near the edge of a street . . . and I’ll get . . . yelled at from a car to use the sidewalk instead of the street. And then . . . if I use the sidewalk . . . I ride by a retirement center and there are people in wheelchairs. The particular sidewalk that I ride along has [a] lady that rides in a wheelchair and then she has her dog on a leash. . . . People don’t understand that just because I’m not in a car doesn’t mean I’m not a person too and I’m entitled to the roadway.

Or, as another participant put it, “While we have great sidewalks inside the neighborhood, once you get to the edge of the neighborhood, it’s like a war zone, because both sides of the street that lead into my neighborhood drop off into a ditch, and if you ride or walk you’re liable to be hit by a car.” This powerful use of stories in addition to data about sidewalks, traffic, and injuries made efforts to pass the Complete Streets resolution personal and meaningful to listeners.

### Communicating with the public about Complete Streets

Other lessons learned through the process of passing Complete Streets involved communicating in the right ways with the right people. For example, participants noted the importance of using local demographic data wherever possible. They also discussed the need to clearly explain the concept of Complete Streets in language accessible to different groups (eg, engineers, professionals, politicians, the public). Furthermore, participants noted that communication strategies need to involve a media advocate and should promote messages of inclusion, that is, how Complete Streets is designed to facilitate safe transportation for all, regardless of mode:

When we were kind of drafting messages, or when I was sharing with people what Complete Streets was about, we were looking for messages of inclusion. . . . This is about providing the same freedom of mobility, the same independence to everyone. Everyone should be able to choose how they get around.

Participants also encouraged the use of public walks or bike rides on carefully planned routes that illustrated both good and bad examples of streets. Such events can highlight existing problems and the need for Complete Streets. Finally, participants suggested that messages make a business case for Complete Streets by emphasizing ways the program can enhance the attractiveness of an area:

Part of what helped it here was chamber people being behind it. Because, okay, if we’re competing with other cities for businesses, the cities we’re competing with have places for families to ride their bicycles. They have places for people to walk. The communities look like a healthy, safe place for families and all activities, and we’ve got to be that way to attract business.

Although Complete Streets policies vary nationwide in the strength and extent of their language, over 490 regional and local areas, 28 states, the Commonwealth of Puerto Rico, and the District of Columbia have adopted or committed to Complete Streets policies (12). As additional state and local advocacy groups seek to introduce Complete Streets to their governing bodies, they may benefit from the lessons learned and advice offered by participants in this study.

Participants had various types of advice they thought would be useful to others. Several noted the importance of choosing the most effective type of legislation and recommended something stronger than a resolution. This advice reemphasizes the importance of having a diverse advisory group, including those with expertise in the law or experience working with decision makers (13). Participants also suggested seeking a plan from the city for accountability regarding implementation of Complete Streets. Others emphasized the importance of starting advocacy efforts early and carefully considering the timing of other city events that may increase or challenge support for Complete Streets. Having representatives from the city or county on the advisory group would aid implementation of these suggestions.

In communication efforts, key informants recommended using messaging that draws clear links between health, wellness, and Complete Streets. Use of evidence-based stories is a powerful means of communicating with policymakers (14). Therefore, incorporating success stories from other communities may be a helpful way to make the case for Complete Streets.

Participants emphasized the importance of strategic use of the news media and the benefit of holding regular meetings to keep all stakeholders involved. These 2 suggestions are echoed in the literature, which cites the benefits of media advocacy and stakeholder engagement (15,16). As public health practitioners and advocates seek to apply lessons learned from Topeka, many tools are available to aid them in determining best evidence, assessing community needs, and planning and evaluating programs (17).

Participants offered additional advice:

- Show examples of communities with Complete Streets; if possible, choose communities that are comparable, close in proximity, or in-state.
- Address all neighborhoods in a community equally to ensure that each has the same opportunity for inclusion in advocacy and outcomes.
- Anticipate the areas of opposition and be prepared to discuss them.
- Keep energy and momentum going, including through the implementation stage.
- Generate and use buy-in from community leaders and grassroots support.

## Interpretation

Evidence is growing about the importance of engaging stakeholder and community groups when attempting policy and environmental change as a means of increasing physical activity and promoting an active lifestyle (18). For example, 1 group of advocates in California used a community-action model to increase local awareness of pedestrian and bicycle safety issues and ultimately bring Complete Streets to Sacramento (19). This group of advocates suggested that part of their success was due to their diversity, as well as their efforts to seek community input from the outset of their work together. Another group in Massachusetts working to encourage active living in everyday life reports, like Topeka participants, that identifying and working with champions of the cause is important, as is planning for sustainability from the beginning of a project (20). Such studies echo lessons learned in Topeka.

Several limitations warrant mention. Although interviews were conducted with supporters of the Complete Streets movement in Topeka, no interviews were formally sought with those opposing it. Thus, the events described only represent the perspective of supporters. Also, every city and local community is different; what worked in Topeka may not necessarily work in other cities. Nonetheless, many lessons learned can be broadly applied in other contexts.

Complete Streets can make a community attractive and safe while making it easier for residents to lead healthy and active lives (6). Lessons learned from the process of passing a Complete Streets resolution in Topeka can apply to other communities. Principal lessons learned involve clearly defining Complete Streets; educating the public, advocates, and decision makers about Complete Streets and how the program enhances a community; building a strong and diverse network of supporters from all sectors; and using stories and examples from other communities with Complete Streets to build a convincing case. This advice is reflected in a summary comment from a Topeka City Council member:

Yeah, well we had a very persuasive one or two people that came to talk to us about it . . . and it was a good, solid, information-packed presentation on the benefits, the economics of it, the safety aspects, the health aspects of being able to walk and bicycle more. . . . That was basically why I supported it, because it was a good case made from end to end by the people that were excited about the concept.
